# Touchless short-wave infrared imaging for dynamic rapid pupillometry and gaze estimation in closed eyes

**DOI:** 10.1038/s43856-024-00572-1

**Published:** 2024-08-06

**Authors:** Omer Ben Barak-Dror, Barak Hadad, Hani Barhum, David Haggiag, Michal Tepper, Israel Gannot, Yuval Nir

**Affiliations:** 1 Department of Physiology and Pharmacology, Faculty of Medical and Health Sciences, Tel Aviv University, Tel Aviv, 6997801 Israel; 2 School of Electrical Engineering, Faculty of Engineering, Tel Aviv University, Tel Aviv, 6997801 Israel; 3 Triangle Regional Research and Development Center, Kfar Qara, 3007500 Israel; 4 Department of Biomedical Engineering, Faculty of Engineering, Tel Aviv University, Tel Aviv, 6997801 Israel; 5 Sagol School of Neuroscience, Tel Aviv University, Tel Aviv, 6997801 Israel

**Keywords:** Sleep disorders, Locus coeruleus

## Abstract

**Background:**

Assessments of gaze direction (eye movements), pupil size, and the pupillary light reflex (PLR) are critical for neurological examination and neuroscience research and constitute a powerful tool in diverse clinical settings ranging from critical care through endocrinology and drug addiction to cardiology and psychiatry. However, current bedside pupillometry is typically intermittent, qualitative, manual, and limited to open-eye cases, restricting its use in sleep medicine, anesthesia, and intensive care.

**Methods:**

We combined short-wave infrared (SWIR, ~0.9-1.7μm) imaging with image processing algorithms to perform rapid (~30 ms) pupillometry and eye tracking behind closed eyelids. Forty-three healthy volunteers participated in two experiments with PLR evoked by visible light stimuli or directing eye movements towards screen targets. Imaging was performed simultaneously on one eye closed, and the other open eye serving as ground truth. Data analysis was performed with a custom approach quantifying changes in brightness around the pupil area or with a deep learning U-NET-based procedure.

**Results:**

Here we show that analysis of SWIR imaging data can successfully measure stimulus-evoked PLR in closed-eye conditions, revealing PLR events in single trials and significant PLRs in nearly all individual subjects, as well as estimating gaze direction. The neural net-based analysis could successfully use closed-eye SWIR data to recreate estimates of open-eye images and assess pupil size.

**Conclusions:**

Continuous touchless monitoring of rapid dynamics in pupil size and gaze direction through closed eyes paves the way for developing devices with wide-ranging applications, fulfilling long-standing goals in clinical and research fields.

## Plain language summary

Monitoring eye movements and pupil size is important for both clinical assessments and in biomedical research. However, measurements are usually made on open eyes. We developed a method that continuously monitors pupil size when eyes are closed and compared it to results obtained when eyes are open. Our approach is contactless and can measure pupil size and gaze direction for hours. This approach has many potential clinical and research uses. For example, it could be used to monitor the extent of arousal during and following anesthesia, sleep, and in unconscious patients.

## Abstract (web summary)

Ben Barak-Dror et al. present a short-wave infrared imaging method for touchless monitoring of pupillary light reflex (PLR) and gaze direction in closed eyes. Data obtained is comparable to that obtained from open eyes.

## Introduction

Evaluation of gaze direction (eye movements), pupil size, and the pupillary light reflex (PLR) plays a crucial role in neurological examination and in other clinical domains^1^ as well as in neuroscience and cognitive research^2,3^. Pupil size is affected by several factors^4–7^. Although a full understanding of these processes remains incomplete, the following main factors play prime roles. First, pupil size is affected by luminance (light levels), where brightness leads to pupil constriction (miosis), and darkness leads to pupil dilation (mydriasis). Pupil constriction in response to bright light is defined as the pupil light response. Although pupil responses represent both reflexive and voluntary actions, the PLR in response to bright light is mainly reflexive, i.e., the same stimulus leads to a qualitatively similar response. Second, pupils constrict in response to near fixation (the pupil near response, or PNR). Third, pupil size is affected by the internal state of brain activity, arousal, and cognition, modulated by both external events and spontaneously. States of anesthesia, sleep, and vigilance are associated with robust changes in pupil siz﻿e^4,8–10﻿]^. In addition, alerting, orienting, and executive functioning are also associated with pupil size dynamics^6^. An orienting response that includes pupil dilation is most pronounced for unexpected salient stimuli such as brief and sudden loud sounds^11^. The relation between arousal and pupil size is mediated by neuromodulatory mechanisms, including the locus coeruleus (LC)^3,12^, which dilates the pupil by inhibiting the parasympathetic Edinger–Westphal nucleus^13^. Accordingly, pupil size provides valuable information about the momentary state of arousal. Functionally, dynamic modulation of pupil size continuously optimizes vision for specific conditions by adjusting sensitivity (e.g., to detect faint stimuli in darkness), regulating visual acuity (smaller pupils increase the sharpness of the image on the retina), and modifying depth of field (small pupils allow sharp vision across a wide range of distances, acting like a small camera aperture). Mechanistically, pupil size is controlled by a dynamic interplay of the parasympathetic constriction pathway with its iris sphincter muscle effector versus the sympathetic dilation pathway with its iris dilator muscle effector^4^.

Pupillometry refers to measuring dynamics in pupil size^4^, which changes spontaneously or in response to bright light that gives rise to the PLR (see^1^ for a detailed review). PLR is evaluated in response to a brief flash of light; typical clinical settings use a flashlight for light exposure, and the eye is observed without dedicated instruments (but see also “Discussion”), whereas in laboratory conditions, a screen is often used for illumination and the eye is monitored via video-based imaging (“eye tracking”). The PLR exhibits a stereotypical time course where multiple parameters (e.g., latency, constriction time, return to baseline) carry important clinical information^1^. In healthy individuals, the PLR is largely symmetric across both eyes^1,14–16^. Even when light stimulation is only delivered to one eye, the response of the eye exposed to light (the “direct response”) has similar temporal dynamics to the eye not exposed to light (the “Consensual response”)^15^, although its constriction amplitude is slightly reduced (see also “Discussion”). Such symmetry is due to PLR engaging bi-lateral constrictive and dilation pathways operating largely reflexively. Different PLR across the two eyes (known as contraction anisocoria) are typically within ±0.2 mm (~<5%) in healthy individuals^17^, but larger differences occur in clinical conditions such as third nerve palsy or Horner’s syndrome^18^.

In the clinic, pupillometry finds applications in numerous domains related to the autonomic system including neurology and head trauma, critical care and emergency medicine, neurosurgery, endocrinology, neurodegeneration, drug addiction, psychiatry, pain, and cardiology^1^. While research settings routinely employ quantitative pupillometry using dedicated hardware and software eye-tracking systems^3,6^, in the clinic pupillometry is typically sporadic and performed manually using a penlight and ruler (but automated pupillometry is gaining some momentum^19^, see also “Discussion”). Therefore, clinical pupillometry is usually time-consuming, inaccurate, subjective, and lacks continuity^20^. Most restricting, pupillometry is limited to situations where the eyes are open^9^. Thus, despite its potential to detect arousal and brain state, pupillometry is not employed during surgical anesthesia or sleep due to unreliable technology for monitoring pupil dynamics behind closed eyelids. Ideally, a medical device would perform touchless quantitative pupillometry continuously to enable applications such as monitoring pain, awareness, or abnormal arousal during anesthesia or sleep. Here, we present a method and system for such a device by showing that touchless short-wave infrared (SWIR, ~0.9–1.7 μm) imaging^21^, combined with dedicated data analysis, can reliably monitor rapid (~30 ms) dynamics in gaze direction and pupil size through closed eyes.

## Methods

### Data acquisition

Experiments were performed on 43 healthy volunteers (ages 19–50, 24 females) in accordance with the institutional review board of the ethical committee at Tel Aviv University (approval 0005827-1) and radiation safety requirements (IEC 62471 international standard, see also Supplementary Note 1). Each volunteer signed an informed written consent for participating in the observational study employing eye tracking taking place between January 2023 and March 2024.

The approach for the main experiment was based on artificially inducing changes in pupil size and pupil position (reflecting gaze direction), measuring the pupil parameters in a closed-eye setting using methods described below, and validating these measurements by comparing them to the concurrent open-eye measurements used as the ground truth. For this purpose, the participants were asked to sit motionless and keep one eye open (right eye) and the other closed (left eye) by holding their finger on the eyelids near the eyelash line. This method relies on the notion that, in healthy individuals, changes in pupil size and gaze are mainly synchronized across eyes^4^.

### Optical setup

**Fig. 1:**
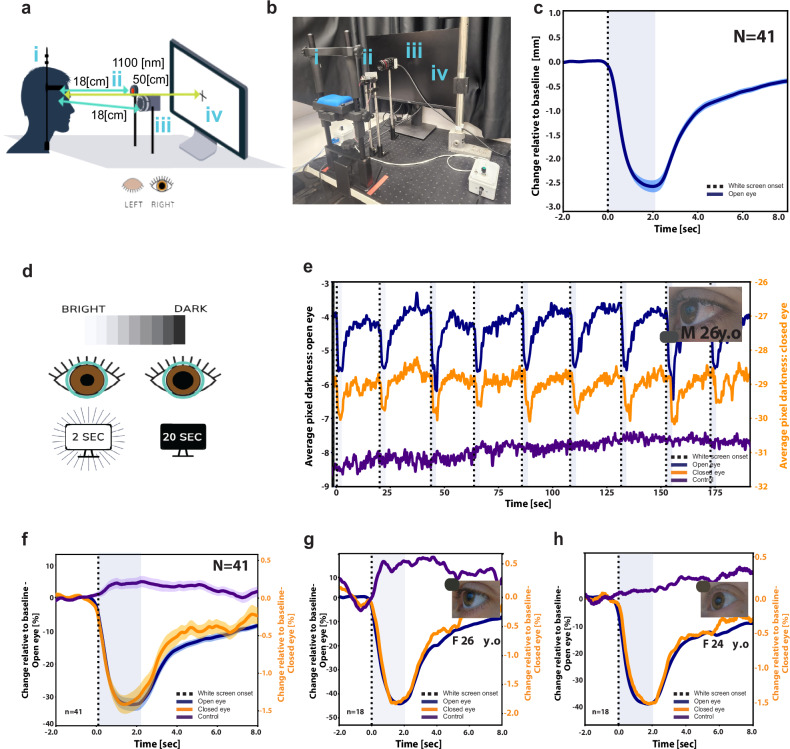
Pupillary light reflex (PLR) assessment in open-eye and closed-eye conditions. **a** Schematic illustration of the experimental setup and **b** actual setup, including (i) chinrest and forehead post used for placing subject face in a fixed position, distance, and angle from illumination sources and cameras, (ii) 1100 nm LED, (iii) SWIR camera and, (iv) screen used to present bright stimuli for PLR measurements. **c** Open-eye PLR as measured with SWIR camera and pupil size extracted with DeepLabCut (*N* = 41). A time course of pupil size (change from baseline in mm, *y*-axis) as a function of time (s, *x*-axis) around brief light stimulation (starting at time zero) reveals typical PLR dynamics. Shading denotes the standard error of the mean across participants. **d** Schematic illustration of the “fixed circle” analysis approach. Pixel darkness (inverse of pixel intensity) is averaged within a fixed circle (teal contour) that roughly captures the pupil and surrounding iris. When the pupil is dilated (right), dark pixels (pupil) comprise a larger percent of the circle, yielding higher average darkness values. Upon light illumination and associated PLR (left), the pupil constricts, and dark pixels (pupil) comprise a smaller portion of the circle, yielding lower average darkness values within the fixed circle. **e** Representative 120 s continuous data of one participant (26 y.o. male, dark brown eyes). Three-time courses depict the average darkness in different regions of interest: blue, open eye; orange, closed eye; purple, control forehead region. **f** Grand average of PLR dynamics (*N* = 41 participants), colors as e. Shading denotes the standard error of the mean across participants. **g**, **h** Two additional examples of average PLR dynamics for participants with different iris colors (26 y.o. female green eyes, 24 y.o. female light brown eyes).

The experimental setup included a chinrest, LED illumination, a SWIR camera (NIT, WiDy SenS 640V-ST), and a computer screen (Fig. 1a, b). Participants sat 50 cm from the presentation screen while their heads rested on a dedicated chinrest (Eyelink®, SR research as in^22^). The participant’s face and eyes were illuminated with an 1100 nm LED (Thorlabs M1100D1, 168 mW(min)) placed 18 cm from the chinrest at an approximate height of the eyes, with a slight angle (~5°) with respect to the axis between the participant and center of the screen. The video was captured with a 31 Hz frame rate via the SWIR camera equipped with a 16 mm lens (LM16HC, Kowa), placed 18 cm from the chinrest directly in front of the subjects at an approximate height of the eyes. Experiments were conducted in a dark room with constant ambient light and a temperature of 24 °C, located in Nir’s lab at Tel Aviv University.

### Experimental design

#### Pupillary light reflex

In the PLR experiment, to measure pupil size dynamics, we focused on light-induced changes in pupil size: a well-studied highly robust phenomenon referred to as the pupillary light reflex^1^. We used a computer monitor (Dell 27 Monitor—P2719H, typical brightness of 300 lux, Dell Inc., Round Rock, TX) placed 50 cm from the participant as the light stimulus (Fig. 1a, b) and controlled its brightness via software (Python 3.6) as described in^23^. Ten visual stimulation trials (uniform white screen, 2 s duration each) were interleaved with inter-stimulus intervals (uniform black screen, 20 ± 2 s duration, so that jitter in timing avoids prediction bias). The experiment lasted a total of 240 s and was repeated twice (with three participants completing only one full experimental session).

#### Gaze direction

In the second experiment, conducted in the same session immediately after the first one, we focused on tracking changes in gaze direction. After a 15 s baseline period, we showed a series of eight crosshair fixation targets on the screen, one at a time. Each target appeared on the screen for 5 s, and the sequence was repeated twice. Subjects were instructed to fixate on these targets, as is customary when calibrating commercial eye trackers before cognitive experiments (e.g.,^22^). The eight positions represented a 3*3 grid apart from the bottom center position (this area of the screen was partially obscured by the camera). The experiment lasted a total of 105 s.

#### Control experiments

**Fig. 2:**
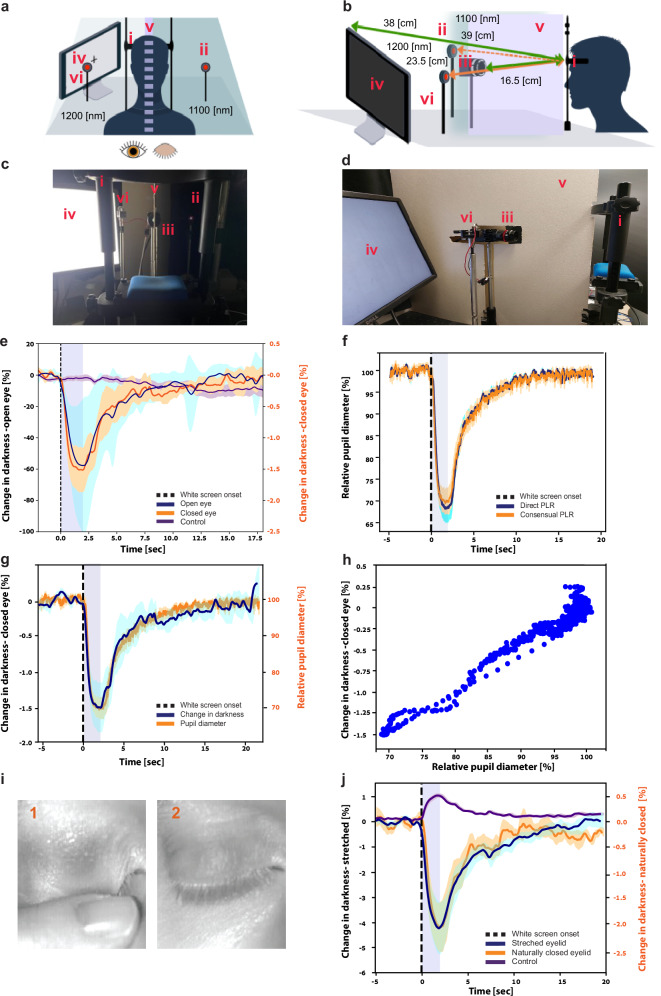
Control experiments establish that closed-eye imaging goes beyond potential confounding factors. **a** Schematic illustration of the experimental setup: view from the back of the subject’s head and **b** as from a side view. Setup elements: (i) chinrest and forehead post used for placing subject face in a fixed position, distance, and angle from illumination sources and cameras, (ii) 1100 nm LED for the consensual side, (iii) SWIR camera, (iv) screen used to present bright stimuli for PLR measurements, (v) 1200 nm LED for the direct side, and (vi) chipboard barrier. **c** Actual setup from the back of the subject’s head, setup elements as above. **d** Actual setup as seen from a side view. **e** Grand average of PLR dynamics with the barrier between both eyes (*N* = 8 participants): blue, open eye in the direct PLR trigger side; orange, closed eye in the consensual (dark) side; purple, control forehead region in the dark side. **f** Grand average of pupil diameter dynamics relative to the baseline pre-PLR diameter with the barrier between both eyes (*N* = 8 participants): blue, open eye in the direct PLR trigger side; orange, open eye in the consensual (dark) side. **g** Comparison of the brightness (left axis, black) and diameter (right axis, orange) changes relative to the baseline, revealing a similar pattern (*N* = 8 participants). **h** Brightness changes relative to baseline dependence on the diameter changes relative to baseline. (*N* = 8 participants). **i** (1) Example of an eyelid held closed with the finger, causing stretching of the eyelid and (2) a naturally closed eyelid of the same participant. **j** Grand average of PLR dynamics with stretched vs naturally closed eyelids (*N* = 6 participants): blue, stretched eyelid; orange, naturally closed eyelid; purple, control forehead region. Shading around time courses in all panels denotes the standard error of the mean across participants.

Following the main set of experiments, additional experiments were conducted separately to estimate the effect of several confounding factors:

Screen light interference (barrier): An experiment was conducted to verify that the observed optical changes were a result of the actual PLR changes and not the changes in the illumination reaching the closed eye from the computer screen. The experimental setup was modified to ensure that the visual stimulation illumination would only reach the open eye by including the following changes: First, a chipboard sheet barrier was placed between the subject’s eyes and the camera, in a way that would prevent light from one side to reach the other and would not obstruct the camera’s view of both eyes (Fig. 2a, b). Since the barrier blocked all light from either side, the previously-used 1100nm-wavelength illumination LED was placed on the side of the closed eye (right eye in this experiment) to mimic the original setting, and a second illumination LED (1200 nm wavelength, Thorlabs M1200D3, 136 mW(min)) was placed on the side of the open, left eye, to allow for pupil size monitoring as the ground truth. The computer screen used for the PLR trigger was placed on the side of the open eye. Light intensity measurements (Lux Light Meter Pro version 2.1.1, developed by Marina Polyanskaya on iPhone 12, Apple Inc., Cupertino, CA) were used to verify that the light did not reach the closed-eye side during the illumination periods (270 lux on the screen-side vs. 0 lux on the closed-eye side). The PLR protocol of the main experiment was used for this experiment as well. The fixed-circle approach was used to determine the darkness changes in the closed eyelids for 8 participants (ages 23–44, 62.5% females).

Pupil diameter/calibration (barrier and two open eyes): Although pupil diameter was assumed to be similar in both eyes, as mentioned above, the closed eye was exposed to a lower illumination level due to the reduced optical transmission of the eyelid. Prior studies have shown that although PLR is simultaneously observed in both eyes, single-eye illumination can result in slightly different pupil diameters on the direct and consensual sides^17^. Therefore, when pupil diameter was estimated based on the closed eye, as described above, it is possible that the actual pupil diameter was slightly different. To quantify the maximal effect of this difference, the barrier experimental setup was used for an additional experiment, using the same PLR trigger protocol, except that both eyes were open throughout the experiment. The experiment had two goals: to quantify the differences in pupil diameters during the PLR events and their potential effect on the measured closed-eye illumination and to explore the ability to create a potential calibration method between closed-eye illumination changes and actual pupil diameter.

Eyelid stretching effect (both eyes closed): During the main experiments, one eye was kept closed while the other eye was open. As it is difficult to maintain this condition throughout the duration of the experiments, the subjects used their finger to hold the eyelid closed. This caused the eyelid to stretch, resulting in a potential reduction of eyelid thickness and consequent artificial increase of the eyelid transparency. In clinical applications, the eyelids are expected to be naturally closed. To verify that the proposed methods work for naturally closed eyes, an experiment was conducted in which the participants (*N* = 6, ages 23–42, 50% females) repeated the PLR experiment protocol in two sessions: one with both eyelids held down using the participant’s finger, and another in which both eyelids were naturally closed.

### Data exclusion

Experimental videos with excessive movement were excluded from subsequent analysis. Since participants used a chinrest during the experiment and held the eyelid with their finger below the pupil area, head and eye movements in the vertical *y*-axis were minimal and were basically limited to the horizontal *x*-axis. To identify and exclude experimental sessions with excessive head movements or eye movements, we extracted the position of the pupil’s center in the open-eye images using DeepLabCut (DLC). For each experimental session separately, we computed the degree of horizontal movement over time (STDt), and set an exclusion threshold at average (STDt) + 1 SD (STDt) across participants (see Supplementary Fig. 1). Eight out of 83 experimental sessions (9.6%) exhibited movement beyond this threshold and were excluded from subsequent analysis.

### Data analysis

In total, 43 individuals participated in the two main experiments. In the PLR experiment, 75 sessions were analyzed following the exclusion criteria mentioned above (*N* = 41 participants). In the second “gaze direction” (crosshair fixation) experiment, analysis was performed on 40 participants.

#### Open-eye validation

To verify we can capture PLR dynamics with our setup and when using a computer screen as the light stimulus, we employed DeepLabCut (DLC), a deep-learning pose-estimation and feature-tracking software, to track changes in pupil size^24,25^ using SWIR video images of the open-eye (Fig. 1c). Pupil diameter was expressed in mm changes relative to the pre-stimulus baseline, as in^23^.

#### “Fixed circle” approach analysis

To quantify PLR dynamics in closed-eye SWIR data via our “fixed circle” analysis approach (Fig. 1d–g), we first defined, for each individual separately, a circle whose position and size approximated the area of the pupil and iris in the closed eye. Pupil position in the closed eye was estimated based on its position just before the eye was closed, using the distance between the pupils (as measured when both eyes were open) or by identification of a darker area behind the closed eyelid. A similar second circle with equal size defined a region of interest (ROI) in the open eye, and a third circle with equal size defined a control ROI on the forehead center. For each circle and time point separately, we averaged the pixel darkness (—brightness value) to create time-courses shown in Fig. 1e. Baseline pupil diameter was defined as the mean pupil diameter during 2 s before light stimulation onset.

#### Removing blink events

Average pixel intensities that exhibited values larger than the average + standard deviation were marked as blinks. To characterize the blinks’ duration, a window of 1 s around all the blinks was extracted and averaged, then the average blink duration was measured at 95% relative height. The values within this calculated blink duration were replaced with linear interpolation of the intensity values before and after the blink. Average pixel intensity data were forward-backward filtered, using a 1 Hz low pass filter (Python function “Filtfilt”—digital filter forward and backward). After this preprocessing, time courses were averaged per participant to show average dynamics per subject (Fig. 1g). A grand average (Fig. 1f) was calculated across all participants, representing 675 (75*9) trials for all ROI.

#### U-NET neural net analysis

We implemented U-net neural net analysis^26^ in Python3.8, ‘Tensorflow2.7’, and ‘Keras’ libraries. Implementing skip connections in the fully convolutional encoder-decoder neural network facilitated the capturing of different latent representations within various network layers. A separate model was trained for each participant. The training was conducted per frame for very short (180 s) videos, using a highly imbalanced dataset due to the short temporal nature of PLR events, comprising only part (~55%) of the experiment’s duration. The data were divided such that two out of the nine trials were used as the test set (PLR events #5 and #7), while the remaining (i.e., 7 PLR events) were used for training. During training, 2 trials (PLR events #4 and #6) were selected as a validation set, and the rest constituted the training set. Two different models were trained for each trial, one closed-eye data and another based on data from the control forehead region. Prior to insertion into the network, each image was normalized by subtracting its mean brightness and dividing by the frame’s standard deviation, with the goal of reducing the effects of global brightness changes between frames. The mean absolute error (MAE) loss was employed for training the network for 200 epochs (a parameter that defines the number of times that the learning algorithm will work through the entire training dataset), with an early stop set to 20 epochs without any improvement on the validation set, across all participants.

After training, predictions for the test set from all participants were used to train a DLC model, which marked the boundaries of the pupil within the eye (e.g., middle square with orange contour in Supplementary Video 1). A time course of the dynamics of the radius of this circle over time was calculated for the ground truth open eye, for the predicted open eye using closed-eye data, and for the predicted open eye using control region data. Blinks in the predictions were filtered (1 Hz lowpass filter) as described above. Performance evaluation was based on MAE across the entire PLR events extracted, starting 2 s before stimulus onset up until 18 s after stimulus onset.

#### Gaze direction estimation

In the closed eye, a dark circle was visible in the SWIR image for nearly all participants at the anticipated position of the closed eye’s pupil. A preliminary assessment of the gaze direction was performed by observing the position of the dark circle in the eye based on DLC tracking. To track changes in the gaze direction with closed eyes, we trained two separate DLC models; one receiving images cropped to include only the open eye, and the other receiving images cropped to include only the data of the closed eye. Gaze direction estimation was then conducted on frames representing the moments when participants were instructed to gaze toward the specified targets.

**Fig. 3:**
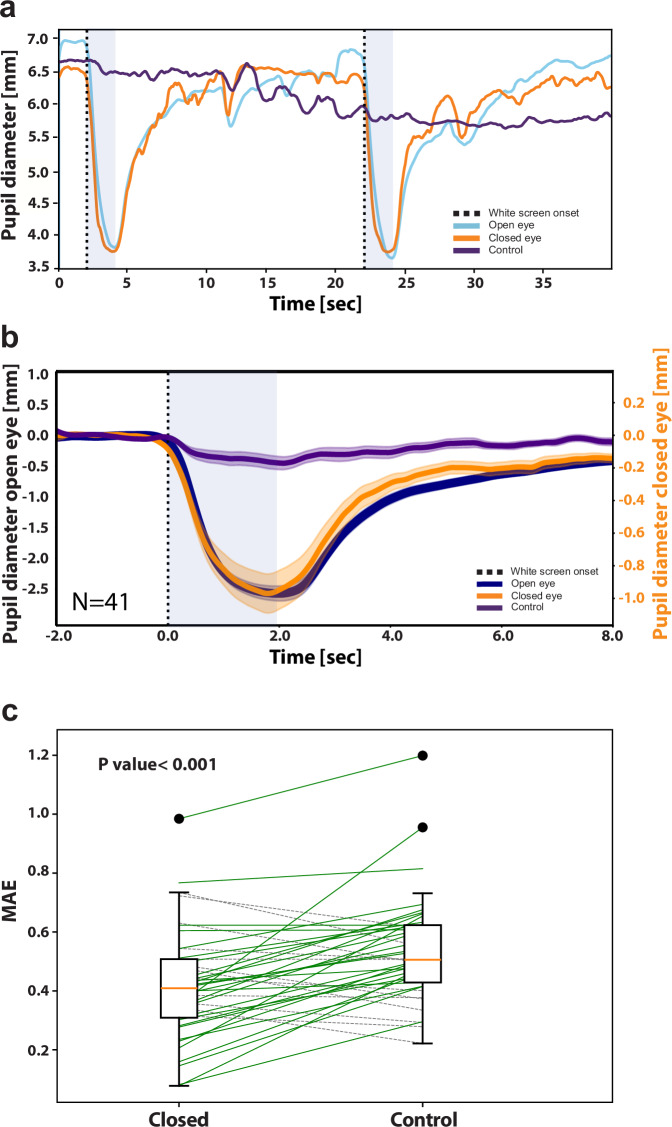
Deep learning analysis of SWIR data captures PLR dynamics through closed eyes. **a** Representative 36 s data of one participant (27 y.o. female, dark brown eyes). includes dynamics around two PLR events (Methods). Three-time courses depict the changes in pupil diameter in the three regions of interest: blue, open eye; orange, closed eye; purple, control forehead region. **b** Grand average of PLR dynamics for all experiments, Three time-courses depict the average darkness in different regions of interest: blue, open eye; orange, closed eye; purple, control forehead region. Shading denotes standard error of the mean across participants (*N* = 41 participants). **c** Mean average error (*y*-axis, *N* = 41 participants) for closed eye (left, median of 0.41 mm, SD = 0.18) and the control region data (right, median of 0.51 mm, SD = 0.18). Each line connects coefficients for the same participant (full line, correlation is higher in closed-eye data; dashed line otherwise). *t*(40) = 4.34, *p* = 9.47e^−05^, 95% CI [0.06, 0.17] via paired two-tailed *t*-test.

Gaze direction estimation was based on identifying changes in horizontal and vertical pupil position relative to central fixation in the image of the eye. To this end, pupil position was calculated as the (x,y) center of the eight points marked on the circumference of the pupil (Fig. 3a). For each eye, the (0,0) origin was defined as the median pupil center position for all frames in which the participant was fixating the central crosshair target. Relative pupil positions were calculated by subtracting the pixel coordinates of the origin from the absolute pupil center positions, for the open and closed eyes separately, based on their respective DLC analyses. Relative pupil positions in pixels were converted to mm using a calibration target included in the imaged field of view.

Finally, changes in pupil position in the image of the eye (mm) were also converted to corresponding changes in degrees of visual angle (DVA) based on the setup’s geometry: For each of the crosshair targets on the screen, the reference viewing angle was calculated based on the screen’s distance from the viewer and the target’s distance on the screen from the central target. Assuming that, on average, participants moved their eyes towards each target when it appeared on the screen, the median open-eye pupil center position for each target could be calculated from the DLC output. Matching the expected viewing angles with the median pupil positions when observing these targets enabled us to calculate a ‘calibration ratio’ between the relative pupil position and the DVA and calculate the interpolated DVA for any pupil position during the experiment. Put simply, using this method, we transformed changes in 2D (pixel/mm) coordinates in the image to changes in DVA between gaze direction and central screen location. Next, we characterized the variability of these differences by computing the average deviation across time, separately for each participant.

### Statistics and reproducibility

Experiments were performed on 43 healthy volunteers. No a-priori sample size calculation was performed, but our sample sizes are greater than those reported in relevant literature^27^. We are confident that the sample size is sufficient since the main findings are highly significant statistically, and can be observed in data of most (93%) individual participants.

In addition, a control forehead region-of-interest allows within-subject analysis to minimize effect of inter-subject variability. Several separate control experiments shown in Fig. 2 replicate the main findings.

Statistical analysis was conducted using the SciPy software library for Python. To assess the validity of the fixed-circle method, the Pearson correlation coefficient, and the corresponding R-squared value were computed to determine the relationship between the open-eye darkness levels (corresponding with the pupil diameter) and the average closed-eye darkness levels of the average PLR event for each participant. To determine the statistical significance of this relationship for each experiment, the Fisher transformation was applied to the Pearson coefficients and R-squared values for all participants, and the transformed values were compared to zero (0) values using a paired two-sided Student’s *t*-test. To assess the validity of the network estimation, the MAE was used to compare the average PLR responses of the open vs. closed eyes and closed eye vs. the control region. A two-tailed paired samples Student’s *t*-test was used to compare the open vs. closed and the closed vs. control MAE values of the network estimation to determine its ability to study pupil dynamics vs. whole image changes. A cutoff *P* value of 0.05 was set to determine statistical significance.

A “*robust PLR*” was defined as a response in which the average pixel darkness at an interval of maximal constriction (1.5–2.5 s after stimulus onset) was significantly lower than the baseline average darkness during the 2 s before stimulus onset (*p* < 0.05 via two-tailed *t*-test).

For the gaze experiment, we calculated the relative difference between the open-eye ground truth and the closed-eye estimation, separately for vertical offsets (*y*-axis) and for horizontal offsets (*x*-axis). Across all participants (*n* = 40), the median MAE of the pupil position estimation was calculated. Then, we compared real data (simultaneous data of closed eye and open eye) to surrogate data (closed eye data vs. open data where 100,000 segments of the experiments were randomly shuffled in time) in order to calculate the statistical significance of the DVA estimation accuracy.

### Reporting summary

Further information on research design is available in the Nature Portfolio Reporting Summary linked to this article.

## Results

### Pupillary light reflex (PLR) assessment in closed eyes

We constructed and validated an experimental setup to assess PLR (Methods, Fig. 1a, b). This setup comprises a chinrest, an infrared (IR) LED source for electromagnetic radiation, cameras, and a computer screen for delivering visible light stimuli. With this imaging setup, continuous imaging can be performed for hours since the IR exposure levels of the illumination conform to safety guidelines without posing risks (see Supplementary Note 1 for detailed safety analysis). To elicit PLR, the screen intermittently presents bright light stimuli (2 s duration, 270 lux near the eye, Methods) at intervals of approximately 20 s, as in^23^. First, we confirmed that the system can reliably capture PLR in open-eye conditions when subjects direct their gaze forward, identifying pupil boundaries with the well-established motion estimation algorithm DLC in video data captured by the SWIR camera (Fig. 1c).

Next, we developed a data analysis method that can work without pupil segmentation. The rationale was that the contrast between pupil borders and the surrounding iris allows for robust segmentation in open-eye conditions. However, we anticipated that the subsequent imaging in closed-eye conditions may not necessarily yield a strong contrast between the pupil and surrounding iris (i.e., sharp pupil borders may not necessarily be observed). To address this challenge, our approach measures the average pixel darkness within a fixed circle that includes the area of both the pupil and surrounding iris (cyan contour, Fig. 1d). When the pupil constricts (e.g., in PLR events), the area of black pixels within this fixed circle reduces, decreasing the average pixel darkness in the circle (Fig. 1d, left).

Next, we assessed PLR in closed-eye conditions using SWIR imaging. Participants held one eye closed throughout two experimental sessions while the other was open. Leveraging symmetry of pupil dynamics across eyes in healthy individuals, we compared closed-eye SWIR imaging to the open-eye data used as ground truth. Figure 1e shows a representative trace of estimated pupil dynamics from closed-eye SWIR data (using “fixed circle darkness” approach, as above) where deflections could be readily observed around each PLR event. Comparing closed-eye SWIR data estimates against the ground truth open-eye measurements (Fig. 1e) revealed that SWIR imaging could accurately capture PLR dynamics through closed eyes (Fig. 1f) and perform well in participants with different iris colors (Fig. 1g, h). Across the entire dataset, with this setup and parameters, a robust PLR (Methods) could be revealed for 92.7% of the participants (Fig. 1f, see also Supplementary Fig. 2 for individual participant data). For all participants, the average Pearson correlation coefficient between the open-eye average PLR event time-course and the closed-eye average PLR event time-courses was 0.57 (range: −0.36 to 0.99, SD = 0.38) with an average R-squared value of 0.47 (range: 0–0.98, SD: 0.37). The difference between the all-participant fisher-transformed Pearson coefficients and zero was statistically significant (*t*(40) = 7.65, *p* = 2.36e^−09^, 95% CI [0.71, 1.22]) and the R-squared values (*t*(40) = 6.71, *p* = 4.733e^−08^, 95% CI [0.5, 0.94]).

### Control experiments rule out potential confounding factors

Next, we ruled out several potential confounding factors with control experiments (Fig. 2). First, we verified that our estimation methods reflect information related to actual pupil imaging rather than possible information from the eyelid illuminated by the visual stimulus around those times (i.e., to make sure momentary reductions in the fixed circle darkness, interpreted as a smaller pupil, do not simply reflect a brighter eyelid). Several aspects indicate this is not the case: (i) The shape of our PLR time-courses was highly asymmetric (sharp constriction followed by gradual re-dilation) whereas the light stimulus was an “ON-OFF” square wave, (ii) PLR effects extended long after the light stimulus terminated (visible light terminates but pupil takes longer to re-dilate), and (iii) a control forehead region also illuminated by the visible light stimulus could not predict pupil dynamics. In addition, (iv) we conducted a control experiment in which a barrier prevented any light from the screen from reaching the closed eye (Fig. 2a–d). In this experiment, a robust average PLR response was observed in the closed eye of all 8 participants (100%, see Fig. 2e for average response), indicating that the changes in the illumination are caused solely by pupil constriction. For all participants, the average Pearson correlation coefficient between the open-eye average PLR event time-course and the closed-eye average PLR time-course was 0.7 (range: 0.24–0.99, SD = 0.29) with an average R-squared value of 0.56 (range: 0.06–0.99, SD: 0.38). The difference between the fisher-transformed all-participant Pearson coefficients and zero was statistically significant (*t*(7) = 3.85, *p* = 0.006, 95% CI [0.48, 2.03]) and the R-squared values (*t*(7) = 3.05, *p* = 0.02, 95% CI [0.21, 1.69]). Thus, critically, closed-eye imaging could detect a robust PLR even when the closed eye was not exposed to the light stimulus. Second, we verified empirically that the direct and consensual PLR responses were indeed similar in amplitude (within ~5% as described previously^17^), verifying that our approach where one open eye served as ground truth for evaluating the closed-eye imaging was adequate (Fig. 2f). As the participant’s eye in the dark side of the barrier was not exposed to light, its pupil diameter, as measured during the experiment with both eyes open, could be used to calculate a calibration function for the darkness changes measured when the eye was closed under the same conditions. As demonstrated in Fig. 2g, the darkness of the fixed circle and the pupil diameter are highly similar and exhibit a linear correlation (*R*^2^ = 0.96; Fig. 2h). Third, we verified that robust PLR imaging in closed-eye conditions could also be achieved when both eyes were closed *naturally* (Fig. 2i, j)—without holding the eye shut with a finger, a procedure that likely resulted in eyelid stretching and favorable conditions for imaging. All participants (*N* = 6, 100%) demonstrated a robust PLR response for both natural-closed and stretched eyelids.

### Deep learning analysis captures PLR dynamics in closed eyes

To complement the intuitive “fixed circle darkness” approach with more advanced data analysis, we employed deep learning-based image processing to identify pupil dynamics in closed-eye SWIR data. We trained a neural net model using U-NET architecture^26^ with pairs of images of open and closed eyes (“training data”). The model’s output (“test data”), based on closed-eye images only, was a set of images, each representing the estimated image of the eye if it were open, from which we could extract pupil size (in millimeters). Model estimates of pupil size derived from closed-eye data (or, alternatively, from a control forehead region) were compared with the ground truth open-eye data. Figure 3a and Supplementary Video 1 show representative examples of the model’s successful estimation of pupil size based on closed-eye SWIR data. Quantitative analysis confirmed successful estimation around PLR events (Fig. 3b). The median MAE between the ground truth and the estimation from the closed eye was approximately 0.40 mm, significantly lower than MAE between the ground truth and estimations based on the control images, 0.52 mm, *t*(40) = 4.32, *p* = 0.0001, 95% CI [0.06, 0.17], via paired two-tailed *t*-test (Fig. 3c). The results indicate that the neural net prediction was significantly improved by using information from the closed eye images that was unavailable in the control region, such as the pupillary changes.

### Estimating gaze direction through closed eyes

**Fig. 4:**
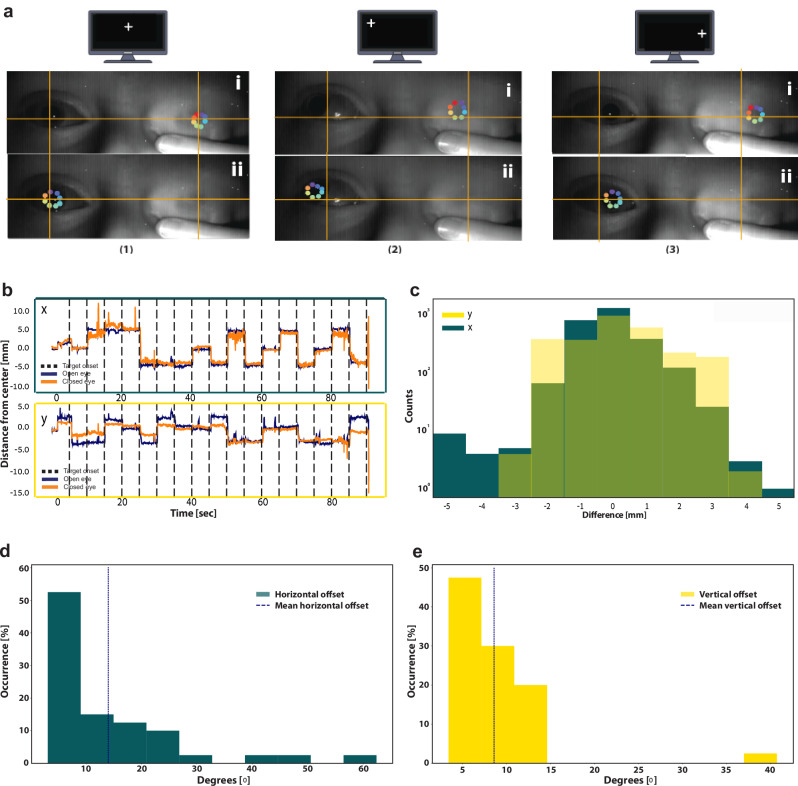
DLC analysis of SWIR data captures gaze direction through closed eyes. **a** Illustration of the DLC gaze tracking procedure: Representative raw SWIR images of open eye (left) and closed eye (right) along with marking of pupil (colored dots) as participants directed their gaze to different screen locations (*N* = 40 participants). The top (i) and bottom (ii) images show pupil positions obtained separately for open and closed eyes, respectively. Orange crosshairs in fixed positions help illustrate differences in pupil position. **b** Typical *x-*axis and y-axis time courses of the pupil distance from the center during the experiment showing a clear distinction of the relative pupil positions as the participant focuses on the different targets (5 s intervals, marked by the dashed lines). blue, open eye; orange, closed eye. **c** The distribution of the relative pupil position differences between the open-eye ground truth and the closed-eye estimation for the same participant, in mm. dark green, *x*-axis (horizontal offset); yellow, *y*-axis (vertical offset). **d** The distribution of the horizontal DVA (degrees visual angle) offset between the open-eye ground truth and the closed-eye estimation for all participants (*N* = 40 participants). Mean error: 14°. **e** The distribution of the vertical DVA offset between the open-eye ground truth and the closed-eye estimation for all participants (*N* = 40 participants). Average error: 8.9°.

Finally, we went beyond pupillometry and conducted a separate experiment that examined *gaze direction* estimation when participants fixated on nine screen positions (Methods, *N* = 40). Gaze direction could be successfully extracted from closed-eye SWIR data (Fig. 4a). To quantify the precision of gaze direction information in closed-eye conditions, we compared ground truth information about pupil position (coordinates obtained from a DLC model applied on open-eye data, Methods) with pupil position coordinates based on closed-eye SWIR data (coordinates obtained from DLC model trained to recognize the dark circle on the eyelid). The relative pupil position was calculated for each eye relative to the pupil position when fixating the central crosshair, serving as the (0,0) origin (Methods). Figure 4b demonstrates a representative example for a single participant, where both horizontal and vertical eye movements toward targets could be readily detected in closed-eye data. Figure 4c shows the distribution of the relative difference between the open-eye ground truth and the closed-eye estimation for this participant, separately for vertical offsets (*y*-axis) and for horizontal offsets (*x*-axis). Across all participants (*n* = 40), the median MAE of the pupil position estimation was 1.4 mm and 3.4 mm for vertical and horizontal offsets, respectively. Finally, we converted the 2D offsets in the image plane of the eye to express them as degrees of visual angle (DVA, see “Methods”) based on our setup’s geometry. This revealed that our gaze direction estimation had typical accuracy reflecting an error of 8.9° and 14° for vertical and horizontal axes, respectively (Fig. 4d, e). The statistical significance of this estimation accuracy was determined by comparing real data (simultaneous data of closed eye and open eye) to surrogate data (closed eye data vs. open data where 100,000 segments of the experiments were randomly shuffled in time). We found that gaze direction estimation was significantly more accurate than that expected by chance in shuffled control data (*p* = 0.000005, d*f* = 100,038).

## Discussion

We have utilized a system and analytical method for contact-free monitoring of rapid (~30 ms) dynamics in pupil size and gaze direction (eye movements) through closed eyes, using a combination of a SWIR imaging system and dedicated data analysis methods. We validate our methods with experiments focusing on PLR in response to visible light stimuli while healthy participants have one eye open (as ground truth) and the other eye closed. With the current setup and parameters used as an initial proof-of-concept, we can already show that a ‘fixed circle darkness’ analysis approach robustly reveals PLR in single trial data and in the vast majority (92.7%) of individual subjects (Fig. 1). Furthermore, deep learning-based methods can reliably predict PLR dynamics through closed eyes (Fig. 3), and gaze direction can be estimated in closed eyes with an error of 8.9–14° DVA (Fig. 4). Our method is, to the best of our knowledge, the first to achieve touchless tracking of rapid changes in pupil size and gaze direction through closed eyes, with great potential in research and clinical care advancements. The PLR, with its robust stereotypical dynamics, was an ideal setting to validate this technology. It can be developed further to track ongoing changes in pupil size and eye movements in many closed-eye domains, including sleep, anesthesia, critical care, and beyond (discussed below).

To characterize the quality and precision of our imaging in a closed-eye condition in a touchless context, we chose to use simultaneously recorded data in the other (open) eye as the benchmark “ground truth”. This is based on the notion that in healthy participants, PLR is largely symmetric (~<5%)^17^. Indeed, our own control experiments confirm that differences between PLR dynamics are within this range (Fig. 2d). While this approach was useful for validation presented here, naturally, we envision that this technology will be applied when both eyes are closed in both research and clinical contexts, and we discuss the milestones towards such translation below.

To the best of our knowledge, only one previous study described an alternative technique for monitoring PLR in closed-eye conditions^27^. The approach in the previous study is based on side illuminating in near-infrared (NIR) through the temple (with light projecting from the back of the eye through the pupil) and imaging through the closed eyelid. Illumination entails brief (0.1 s) pulses 2 cm from the skin at the temple. Due to the proximity of NIR LED illumination to the subject’s skin in this approach, only two brief pulses were used (before and after inducing the PLR). Thus, while this technology may be used for intermittent PLR assessment for very specific clinical applications (e.g., periodic evaluation of pupil reactivity after traumatic transtentorial herniation), it is insufficient for nearly all other applications. By contrast, the current SWIR-based imaging, with its distant (20 cm or higher) 1100 nm LED illumination, can operate continuously in accordance with safety guidelines (IEC 62471). Beyond continuous operation, our approach offers several additional advantages: (i) illumination is positioned away from the face, essential for uninterrupted use without inducing heat, (ii) touchless functionality, vital for tracking natural sleep, and (iii) enhanced performance due to the deeper penetration capabilities of SWIR, which is crucial for sub-second temporal resolution. Tracking the precise time-course of PLR responses with high temporal resolution is key since multiple PLR time-course parameters (e.g., latency, constriction time, return to baseline) carry important clinical information^1^. The average darkness during a PLR event, as tracked by our ‘fixed circle approach’, exhibits a similar time-course profile as the pupil diameter (as seen in Fig. 2e), potentially allowing for a similar calculation methodology of these parameters. Thus, future improvements in the optical setup achieving higher sensitivity and increased resolution should facilitate systematic estimation of these parameters in closed-eye settings. In addition, continuous monitoring with high temporal resolution allows the detection of brief spontaneous changes in pupil diameter that may occur at unexpected times (e.g., pain, seizure, following cardiac arrest, nightmare) indicating abnormal arousal during natural sleep, reflecting changes in anesthesia depth, and beyond.

Automated quantitative pupillometry is gaining momentum and is increasingly being used for various clinical implications. While PLR examination has long been a standard element of the neurological exam for patients with known or suspected neurologic injury, recent data support the utility, reliability, and predictive value of PLR data from *automated* pupillometry^19^, which is being increasingly adopted as part of neurological examination. Handheld automatic pupillometers measure PLR-related values such as constriction velocity, maximum constriction, and latency to derive a “neurological pupil index” (NPi)^28^. Accumulating data demonstrates that automated pupillometry can outperform manual penlight pupil examination in neurocritical care and other clinical domains (reviewed in^19,29,30^). Automated pupillometry can be beneficial in contexts of traumatic brain injury (TBI)^31^, to detect transtentorial herniation (TTH) and increased intracranial pressure (ICP)^32^, diagnose third (oculomotor) nerve palsy^33^ and Horner’s Syndrome^34^, in stroke^35^, for prognosis after cardiac arrest^36^, in critically ill patients^30^, and with respect to effects of numerous drugs ranging from opioids through hypnotics, sedatives, and anesthetics to stimulants and antidepressants^29,30^. Our technology, allowing continuous touchless pupillometry in closed-eye conditions, could expand these applications along several dimensions. First, they allow pupillometry and PLR to be assessed at multiple time points, going beyond a single assessment around neurological examination. In some cases (e.g., life-threatening TTH) early identification of a unilateral pupil dilation without reactivity can be critical for survival^37^. In addition, during anesthesia and natural sleep pupil size provides valuable information in addition to other autonomic measures such as heart rate or blood oxygen saturation (SpO_2_) that are routinely monitored. For example, during anesthesia, noxious stimuli can dilate the pupil even when little or no changes are observed in heart rate^38^. During rapid eye movement (REM) sleep, pupils constrict while breathing, and heart rates are elevated^39^. Thus, our technology could help identify abnormal arousals in contexts of pain and intraoperative awareness during surgical anesthesia^40^, as well as insomnia associated with “restless REM” sleep^41^ and potential biomarkers for autonomic disorders^42^, nightmares, or nocturnal seizures during sleep.

In terms of gaze direction estimations, our approach is based on identifying the location of the pupil’s center in SWIR images using DLC tracking. In our experiments on healthy individuals, we assumed that gaze is conjugated and coordinated across both eyes when fixating at different screen locations (“Methods”). Accordingly, the confirmed location of the pupil’s center in the open eye was used as ground truth to quantify the quality of estimation based on closed-eye SWIR imaging analysis. With this approach, the mean discrepancy between ground truth open eye data and the closed eye across time was up to 14 degrees of visual angle (DVA). This resolution, while not capturing small (micro-) saccades prevalent in open-eye wakefulness conditions, is highly adequate to monitor the prevalence, amplitude, and trajectories of eye movements occurring in closed-eye conditions, as is the case, for example, when scoring REM sleep^43^, or when detecting roving eye movements in a comatose patients^44^. Future improvements to the system, such as advanced algorithms for pupil and iris detection based on brightness patterns or neural net learning are expected to further improve the accuracy of gaze direction monitoring in closed-eye conditions. Gaze direction estimation in closed-eye settings can find several clinical applications such as neurological evaluation in intensive care units^45^, monitoring of anesthetic depth^46^, and non-contact identification of rapid eye movement sleep^47^.

In this study, we separated the assessment of pupil size and gaze direction in two experiments. Thus, the use of the ‘fixed circle darkness’ approach assumed that the pupil remained in the same position throughout the monitoring period. Clinical real-world applications cannot be based on the assumption that the pupil’s position is known. Although the fixed circle approach can be applied to the entire eye area and eliminate the need for pupil position detection, ultimately we envision that accurate gaze detection algorithms can be used to identify narrower regions of interest and increase pupil diameter estimation accuracy. In other words, future work should integrate pupil size and gaze direction estimations.

We anticipate that our method has great potential for improving the monitoring of depth of sedation, analgesia, and clinical changes while under anesthesia^48^. During surgery, clinicians opt to ensure an adequate degree of sedation and analgesia, while avoiding overly deep anesthesia that may jeopardize hemodynamic and respiratory stability^49^ and could lead to adverse post-operative consequences ranging from nausea to delirium in the elderly^50^. In this context, there is increasing interest in noninvasive neuro-monitoring. Above and beyond standard measures such as heart rate, respiratory rate, and pulse oximetry, monitoring of anesthesia depth can be complemented using electroencephalography (EEG), with associated indices such as the bispectral index (BIS)^51^ and the entropy module, or employing evoked potential approaches. Additional methods are based on near-infrared spectroscopy (NIRS) providing noninvasive measurement of cerebral regional oxygenation, and Transcranial Doppler used in the perioperative settings^49^. The method presented here of continuous touchless monitoring of gaze direction and pupil size holds promise in the context of depth of anesthesia monitoring. Pupillometry can be more sensitive to reflect noxious stimulation during anesthesia than the autonomic measures typically monitored, such as heart rate and blood pressure^38^, and does not require contact with the subject (as is the case with EEG).

What are the required milestones for translating this technology into a useful clinical tool? Some current limitations should be explicitly acknowledged, along with associated contingency plans for clinical translation. First, here, we mostly perform imaging with one open eye (as ground truth) and the other eye closed, whereas clinical settings would require imaging of two closed eyes. We provide initial data that imaging can indeed be achieved when both eyes are shut. Furthermore, we show that such imaging is successful without a finger holding the eye shut (as employed in the main experiment here), which could potentially introduce eyelid stretching, and layering (Fig. 2h). Still, our current U-NET deep learning-based analysis (Fig. 3) currently uses each participant’s open-eye data as training for their closed eye. Future work should improve analysis to achieve unsupervised learning that generalizes across individuals. In such a case, an imaging device could monitor pupil size and gaze direction in an individual not previously encountered who has both eyes closed. Second, the current system works in chin-rest conditions that ensure minimal head movements, and participants were instructed to direct their gaze forward. Future work should generalize the system to encompass head movements and integrate gaze direction and pupil dilation measurements to monitor dynamics in pupil size while eye movements occur. In terms of head movements, imaging during natural sleep represents a more ambitious goal than during anesthesia and ICU when posture is relatively still. Third, the current temporal resolution is dictated by the SWIR camera frame capture rate. The current study was conducted with a temporal resolution of ~30 ms. While this is sufficient to capture pupil dynamics that occur at the timescale of tens of milliseconds^5^, a higher temporal resolution may be required to capture fast (<10 ms) gaze changes such as micro-saccades^52^. The SWIR camera used in this study has a temporal resolution of 5 ms with shorter integration time intervals and could potentially be used for this purpose. Further studies are required to study the ability to accurately capture these short-scale changes with this or other cameras. Fourth, any gaze direction estimates based on the detection of pupil center position may be affected by the pupil-size artifact: whereby the pupil isn’t exactly circular and does not enlarge or constrict strictly circularly^53,54^. This limitation is common to most video-based eye-tracking algorithms. Nonetheless, further studies should explore the potential extent of this effect on the gaze estimation accuracy using the SWIR camera. Fifth, our focus so far has been on PLR to leverage its stereotypical time course to provide the most convincing proof that our method can capture pupil size dynamics through closed eyes. Extending the system to *spontaneous* changes in pupil size, which reflect momentary dynamics in arousal, will require analysis of ongoing measurements without averaging across repeated trials and going beyond changes due to fluctuations in ambient light. In addition, arousal-associated pupil dilations could also be of smaller amplitude compared with the PLR constriction. Further improvements in both data acquisition (optical imaging) and data analysis (refining neural net architectures and training models on more comprehensive data) should improve sensitivity to reliably detect smaller spontaneous pupil size changes in real-time and ensure robust performance that is invariant to precise imaging conditions. For some applications (e.g., PLR tests at ICUs) rapid translation can be expected, and clinical trials at patients’ bedside can begin as soon as an operational prototype passes safety and ethical clearance. As we continue to optimize imaging and data analysis, this approach is expected to allow monitoring of continuous variations in pupil size, beyond the PLR.

### Supplementary information

Supplementary Information

Description of Additional Supplementary Files

Supplementary Data 1

Supplementary Video 1

Reporting Summary

## Data Availability

Code supporting data analysis were made available to referees during peer-review to ensure scientific validity and rigor of data analysis and scientific findings. Code supporting data analysis could be made available from the corresponding author (Yuval Nir, ynir@tauex.tau.ac.il) upon reasonable request i.e., adhering to proprietary constraints.

## Data availability

The numerical results (source data) underlying Figs. 1c, e–h, 2e–h, j, 3a–c and 4b–e can be found in Supplementary Data 1. Data supporting the results reported in the article are available from the corresponding author (Yuval Nir, ynir@tauex.tau.ac.il) upon reasonable request i.e., adhering to confidentiality and proprietary constraints.
