# Supplementary material for: Touchless short-wave infrared imaging for dynamic rapid pupillometry and gaze estimation in closed eyes
Source: Commun Med (Lond). 2024 Aug 6;4:157. doi: 10.1038/s43856-024-00572-1 (PMC11303404; doi:10.1038/s43856-024-00572-1)
Supplement: Supplementary file 1 — Supplementary Information [file 43856_2024_572_MOESM1_ESM.docx]

**Supplementary material for**

**Touchless monitoring of rapid dynamics in pupil size and gaze direction through closed eyes via short-wave infrared (SWIR) imaging**

Omer Ben Barak-Dror^1^, Barak Hadad^1^, Hani Barhom^2,3^ , David Haggiag^4^, Michal Tepper^4^, Israel Gannot^4,*^, Yuval Nir^1,4,5,*^

1. Department of Physiology and Pharmacology, Faculty of Medicine, Tel Aviv University, Tel Aviv 699780, Israel

2. School of Electrical Engineering, Faculty of Engineering, Tel Aviv University, Tel Aviv 6997801, Israel

3. Triangle Regional Research and Development Center, Kfar Qara, 3007500, Israel

4. Department of Biomedical Engineering, Faculty of Engineering, Tel Aviv University, Tel Aviv 6997801, Israel

5. Sagol School of Neuroscience, Tel Aviv University, Tel Aviv 6997801, Israel

*Corresponding authors: Yuval Nir, ynir@tauex.tau.ac.il; Israel Gannot, gannoti@tauex.tau.ac.il

**Supplementary Note 1 - Safety and IR exposure**

The experimental setups in this study include two illumination LEDs: One illuminating at 1100nm (Thorlabs M1100D1*,* 168mW(min)) that was used for all the experiments and a second illumination LED used only for the open eye in the barrier experiments (1200nm, Thorlabs M1200D3*,* 136mW(min)). These LEDs were used throughout experiments lasting several minutes and constitute an exposure level that is safe for longer continuous exposures (hours) when used in future clinical settings.

According to the manufacturer, the risk group of both LEDs is classified as “RG0 – exempt” according to the Standard IEC 62471:2006, Photobiological Safety of Lamps and Lamp Systems, indicating that they are safe for continuous exposure at distances of 20cm or more. As the 1100nm LED was used at a distance of 18cm from the participant’s eyes, a full IR exposure analysis was conducted as follows:

Near infrared illumination is associated with three main potential health risks: thermal skin burns, damage to the cornea (leading to cataract), and retinal burns. The following calculations demonstrate the safety of the selected LEDs for each of these risks according to standard guidelines and practices in the context of continuous and extended duration use (ICNIRP Guidelines on Limits of Exposure to Incoherent Visible and Infrared Radiation, International Commission on Non-Ionizing Radiation Protection).

Detailed estimations:

1. Thermal skin burns

According to the literature, for exposures longer than ten seconds, acute pain occurs before the skin can be damaged. In addition, the skin exposure limit is not lower than the cornea exposure limit for exposures of up to several seconds. Therefore, for the purposes of this analysis, it is assumed that the limiting factors are the retinal and corneal exposure limits and not the skin damage limit.

1. Exposure limit for cornea damage

The maximal exposure threshold is the threshold for exposures longer than 1000 seconds. In these scenarios, the exposure limit does not depend on the duration of the exposure and a fixed value is used. For a single source, this value is equal to 100W/m^2^.

At an illumination distance of 18cm, the illumination of the selected LED is $22.2\frac{\mu W}{{mm}^{2}}=22.2\frac{W}{m^{2}}$, which is lower than the $100\frac{W}{m^{2}}$ threshold. Therefore, the used LED is safe considering the cornea exposure limit for continuous use.

1. Exposure limit for retinal damage

For an infrared light source that does not provide a strong visual stimulus, the participants do not avoid the light by movement or pupil constriction. The weighted radiance limit is given by the following formula:

1. $L_{IR}=\sum_{\lambda=780}^{1400} L_{\lambda}\cdot R(\lambda)\cdot\Delta\lambda\leq\frac{6300}{\alpha}\left[ \frac{W}{m^{2}\cdot sr} \right]$

Where α is the source size in radians, with minimal and maximal values, as specified in the referenced literature^1^.

R(λ) is the burn hazard weighing ratio, given by the following formula:

1. $R\left( \lambda\right)={10}^{\left[ \frac{700-\lambda}{500} \right]}$

The spectral radiance of a given source can be approximated using the following formula:

1. $L_{IR}\approx I_{e}\cdot\frac{R(\lambda)}{\left( \left( l+w \right)/2 \right)^{2}}$

For the LED in the setup, the width and length are both 1mm. α is therefore equal to 0.005 and replaced with the minimal effective value of 0.011.

R(1100nm) is equal to approximately 0.16.

The spectral radiance limit is equal to:

(4) $L_{IR\_Limit}\leq\frac{6300}{\alpha}\left[ \frac{W}{m^{2}\cdot sr} \right]\cong572,727\left[ \frac{W}{m^{2}\cdot sr} \right]$

The actual spectral radiance is equal to:

(5) $L_{IR}\approx I_{e}\cdot\frac{R\left( \lambda\right)}{\left( \frac{\left( l+w \right)}{2} \right)^{2}}=\left( 22.2\frac{W}{m^{2}}\cdot\left( 0.18m \right)^{2} \right)\frac{0.16}{{0.001}^{2}}\cong115,200\left[ \frac{W}{m^{2}\cdot sr} \right]$

Since L_IR_ is significantly lower than L_IR_Limit_, this LED is considered as safe in terms of retinal damage.

Therefore, as the LED was found to be safe according to all three relevant aspects of eye exposure, it is considered to be safe for the experiments conducted as well as future continuous use applications.

Importantly, it should also be noted that these guidelines and estimations are for **open-eye settings**. However, in a future clinical setting the illumination will be used on patients with closed eyelids. This will further reduce the intensity of the light reaching the eye, providing an additional layer of protection. In other words, the current calculations represent an over-conservative upper bound for any exposure in future clinical settings.

**Supplementary Figure 1. Data exclusion protocol**

**
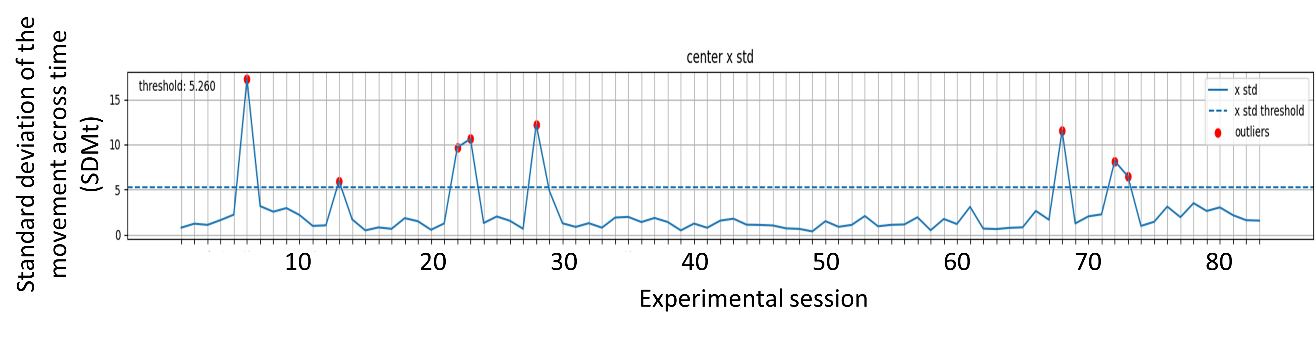
**

Figure Supplementary 1. Exclusion of experiment videos from analysis. All videos in which the x-axis SDM (standard deviation of the movement) as measured in number of pixels were above the threshold (average SDM across all experiments + the standard deviation) were excluded.

**Supplementary Figure 2. Individual participant data**


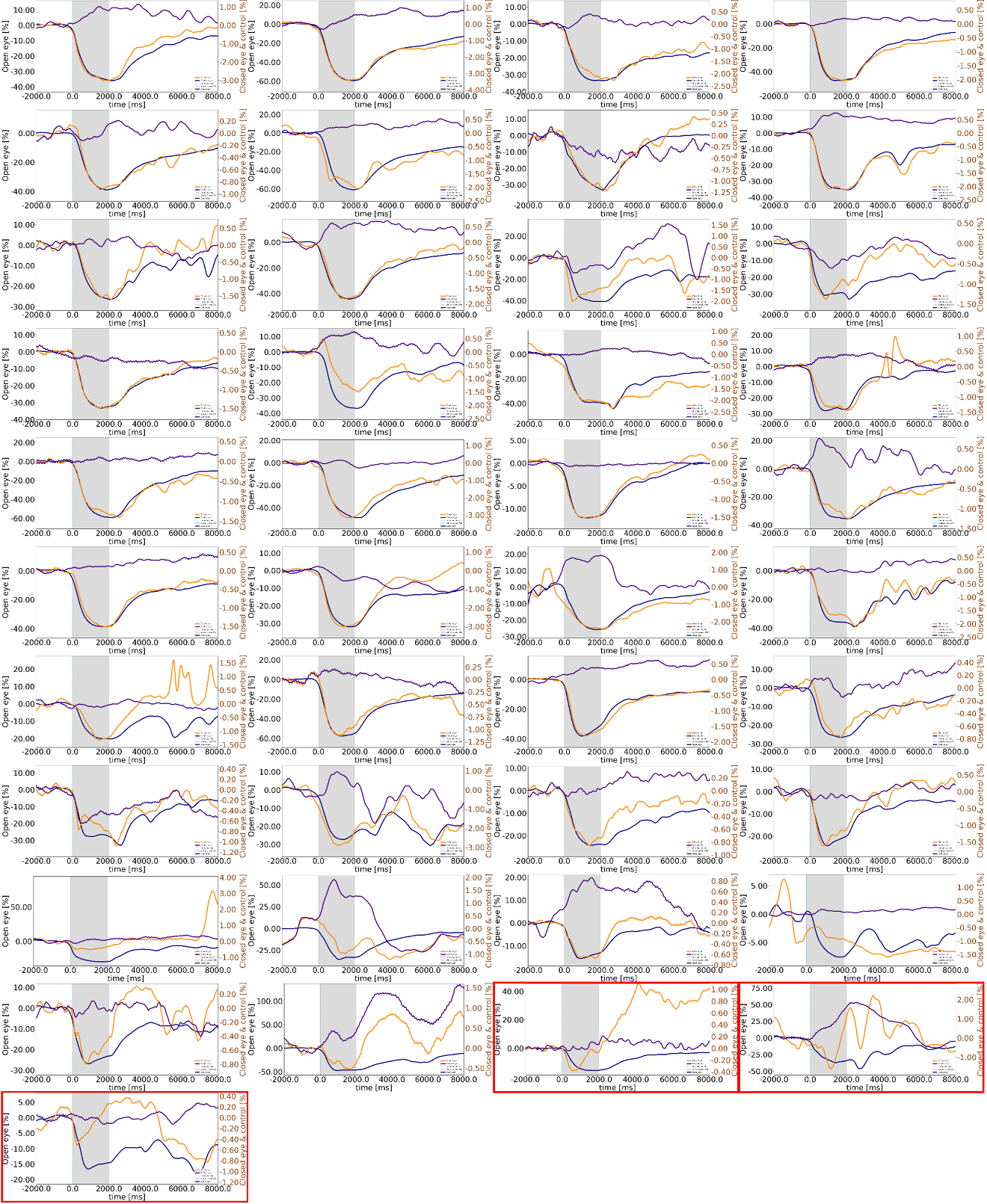


Figure Supplementary 2. Individual PLR responses according to the fixed circle analysis (average pixel darkness, averaged across events, for each participant separately). Blue, open eye; Orange, closed eye; Purple, control forehead region. Note that y-axis (% change relative to baseline) is adjusted separately for open eyes (left, blue) and jointly for closed eye/control (on right, dark orange). Individual participants are ordered (left to right, then row below) by significance of their PLR response (p-value of t-test between estimated pupil size at 1.5-2.5s compared with 2s baseline before stimulus onset; ‘best PLR’ is on top left). Figures representing participants who did not demonstrate a significant decrease in darkness are surrounded by a red frame.

**References**

1. International Commission on Non-Ionizing Radiation Protection. ICNIRP Guidelines on Limits of Exposure to Incoherent Visible and Infrared Radiation. Health Physics 105(1):p 74-96, July 2013. | DOI: 10.1097/HP.0b013e318289a611
