# Supplementary material for: Touchless short-wave infrared imaging for dynamic rapid pupillometry and gaze estimation in closed eyes
Source: Commun Med (Lond). 2024 Aug 6;4:157. doi: 10.1038/s43856-024-00572-1 (PMC11303404; doi:10.1038/s43856-024-00572-1)
Supplement: Supplementary file 2 — Description of Additional Supplementary Files [file 43856_2024_572_MOESM2_ESM.pdf]

Supplementary data 1. Summary of the data from PLR and gaze experiments.

Source data for all 43 participants underlying figures, separately for the two analysis methods described in the article: based on the intuitive 'fixed circle' analysis approach (Figures 1,2), the UNET analysis (Figure 3), and gaze estimation (Figure 4). Data are organized in sheets according to each corresponding figure panel.

Supplementary Video 1. Representative model prediction of open eye images based on closed eye data or control forehead data.

Representative 40s data of one participant includes dynamics around two PLR events (black dashed vertical line denotes white screen onset). Three time-courses depict the pupil size: open eye ground-truth data (cyan), the model's output estimation when trained on closed eye data (orange), the model's output when trained on control forehead region data (purple). High similarity between closed eye estimation and ground-truth is not evident when training on forehead data (Pearson correlations; Closed eye: R=0.986, Control: R=-0.05).
