# Supplementary material for: Touchless short-wave infrared imaging for dynamic rapid pupillometry and gaze estimation in closed eyes
Source: Commun Med (Lond). 2024 Aug 6;4:157. doi: 10.1038/s43856-024-00572-1 (PMC11303404; doi:10.1038/s43856-024-00572-1)
Supplement: Supplementary file 5 — Reporting Summary [file 43856_2024_572_MOESM5_ESM.pdf]

nature portfolio

Corresponding author(s): Yuval Nir, Israel Gannot

Last updated by author(s): 2023/10/12

Reporting Summary

Nature Portfolio wishes to improve the reproducibility of the work that we publish. This form provides structure for consistency and transparency in reporting. For further information on Nature Portfolio policies, see our Editorial Policies and the Editorial Policy Checklist.

Statistics

For all statistical analyses, confirm that the following items are present in the figure legend, table legend, main text, or Methods section.

n/a	Confirmed	
☐	☒	The exact sample size (*n*) for each experimental group/condition, given as a discrete number and unit of measurement
☐	☒	A statement on whether measurements were taken from distinct samples or whether the same sample was measured repeatedly
☐	☒	The statistical test(s) used AND whether they are one- or two-sided<br>*Only common tests should be described solely by name; describe more complex techniques in the Methods section.*
☐	☒	A description of all covariates tested
☒	☐	A description of any assumptions or corrections, such as tests of normality and adjustment for multiple comparisons
☐	☒	A full description of the statistical parameters including central tendency (e.g. means) or other basic estimates (e.g. regression coefficient) AND variation (e.g. standard deviation) or associated estimates of uncertainty (e.g. confidence intervals)
☐	☒	For null hypothesis testing, the test statistic (e.g. *F*, *t*, *r*) with confidence intervals, effect sizes, degrees of freedom and *P* value noted<br>*Give P values as exact values whenever suitable.*
☒	☐	For Bayesian analysis, information on the choice of priors and Markov chain Monte Carlo settings
☒	☐	For hierarchical and complex designs, identification of the appropriate level for tests and full reporting of outcomes
☒	☐	Estimates of effect sizes (e.g. Cohen's *d*, Pearson's *r*), indicating how they were calculated

Our web collection on statistics for biologists contains articles on many of the points above.

Software and code

Policy information about availability of computer code

Data collection	We used custom code (Python 3.8) which is described in the methods section<br>We will share the code for stimulus presentation (screen brightness control) upon reasonable request.
Data analysis	Analysis was performed using custom code (Python 3.8) which is described in the methods section, and represents a combination of novel algorithms and existing custom toolboxes (e.g. 'Tensorflow2.7' and "keras' libraries for U-net neural net analysis). Code has been made available to editors and reviewers to allow peer-review and ensure scientific validity and rigor of analysis pipeline.

For manuscripts utilizing custom algorithms or software that are central to the research but not yet described in published literature, software must be made available to editors and reviewers. We strongly encourage code deposition in a community repository (e.g. GitHub). See the Nature Portfolio guidelines for submitting code & software for further information.

Data

Policy information about availability of data

All manuscripts must include a data availability statement. This statement should provide the following information, where applicable:

- Accession codes, unique identifiers, or web links for publicly available datasets
- A description of any restrictions on data availability
- For clinical datasets or third party data, please ensure that the statement adheres to our policy

Data supporting the results reported in the article are available from the corresponding author on reasonable request.

Human research participants

Policy information about studies involving human research participants and Sex and Gender in Research.

Reporting on sex and gender	We report self-reported gender of 43 participants (24 women, 19 men, 0 other) in the Results and Methods sections. We did not perform gender-based analysis.
Population characteristics	Experiments were performed on 43 healthy volunteers (ages: 19-50, 24 females)
Recruitment	Subject recruitment involved ads on Tel Aviv University campus per institutional ethical committee guidelines.
Ethics oversight	TAU Institutional Review Board (approval 0005827-1)

Note that full information on the approval of the study protocol must also be provided in the manuscript.

Field-specific reporting

Please select the one below that is the best fit for your research. If you are not sure, read the appropriate sections before making your selection.

☒ Life sciences ☐ Behavioural & social sciences ☐ Ecological, evolutionary & environmental sciences

For a reference copy of the document with all sections, see nature.com/documents/nr-reporting-summary-flat.pdf

Life sciences study design

All studies must disclose on these points even when the disclosure is negative.

Sample size	Experiments were performed on 43 healthy volunteers. No a-priori sample size calculation was performed, but our sample sizes are greater than those reported in similar previous publications focusing on methodological proof-of-concept, which often describe one or two participants. We are confident that the sample size is sufficient since the main findings are highly significant statistically, and can be observed in data of most individual participants. In addition, a control forehead region-of-interest allows within-subject analysis to minimize effect of inter-subject variability.
Data exclusions	Data exclusions are explicitly detailed in the Methods (detailed also in Supplementary Figure S1). 8 out of 83 experimental session (9.6%) were not analyzed further due to excessive participant movement, employing a uniform quantitative criterion.
Replication	The main findings (Figure 1) are highly significant statistically and can be observed in data of most (93%) individual participants. Several separate control experiments shown in Figure 2 replicate the main findings.
Randomization	N/A
Blinding	N/A

Reporting for specific materials, systems and methods

We require information from authors about some types of materials, experimental systems and methods used in many studies. Here, indicate whether each material, system or method listed is relevant to your study. If you are not sure if a list item applies to your research, read the appropriate section before selecting a response.

Materials & experimental systems

n/a	Involved in the study	
☒	☐	Antibodies
☒	☐	Eukaryotic cell lines
☒	☐	Palaeontology and archaeology
☒	☐	Animals and other organisms
☒	☐	Clinical data
☒	☐	Dual use research of concern

Methods

n/a	Involved in the study	
☒	☐	ChIP-seq
☒	☐	Flow cytometry
☒	☐	MRI-based neuroimaging
